# Autosomal Dominant Alzheimer Disease: A Unique Resource to Study CSF Biomarker Changes in Preclinical AD

**DOI:** 10.3389/fneur.2015.00142

**Published:** 2015-06-29

**Authors:** Suzanne Elizabeth Schindler, Anne M. Fagan

**Affiliations:** ^1^Department of Neurology, Knight Alzheimer’s Disease Research Center, Hope Center for Neurological Disorders, Washington University School of Medicine, St. Louis, MO, USA

**Keywords:** cerebrospinal fluid, biomarkers, Alzheimer disease, autosomal dominant, familial

## Abstract

Our understanding of the pathogenesis of Alzheimer disease (AD) has been greatly influenced by investigation of rare families with autosomal dominant mutations that cause early onset AD. Mutations in the genes coding for amyloid precursor protein (*APP*), presenilin 1 (*PSEN-1*), and presenilin 2 (*PSEN-2*) cause over-production of the amyloid-β peptide (Aβ) leading to early deposition of Aβ in the brain, which in turn is hypothesized to initiate a cascade of processes, resulting in neuronal death, cognitive decline, and eventual dementia. Studies of cerebrospinal fluid (CSF) from individuals with the common form of AD, late-onset AD (LOAD), have revealed that low CSF Aβ42 and high CSF tau are associated with AD brain pathology. Herein, we review the literature on CSF biomarkers in autosomal dominant AD (ADAD), which has contributed to a detailed road map of AD pathogenesis, especially during the preclinical period, prior to the appearance of any cognitive symptoms. Current drug trials are also taking advantage of the unique characteristics of ADAD and utilizing CSF biomarkers to accelerate development of effective therapies for AD.

## Introduction

In 1901, Dr. Alois Alzheimer began treating Auguste D., a 51-year-old woman with memory loss and hallucinations. Ms. D’s dementia progressed and she died at the age of 56. Upon histopathological examination, Alzheimer found two types of abnormalities in the brain that were later termed amyloid plaques and neurofibrillary tangles (1). Over a century later, when patients die with a characteristic history of progressive cognitive decline and upon autopsy are found to have significant quantities of amyloid plaques and neurofibrillary tangles, they are assigned the neuropathological diagnosis of Alzheimer disease (AD). The vast majority of patients with AD develop dementia at age 65 or older. Genetic studies of patients like Ms. D, who develop cognitive decline before age 65, have revealed rare autosomal dominant mutations that cause AD (2). Recently, surviving samples from Ms. D were subjected to genetic analysis and found to have a genetic mutation in presenilin 1 (*PSEN-1*) (3), although there has been some controversy about this finding (4).

There is some concern that the pathogenesis of autosomal dominant AD (ADAD) may vary from the common late-onset AD (LOAD). However, while there are certainly some differences between ADAD and LOAD in terms of disease etiology, clinical features, and neuropathology, they share many characteristics including an abnormal pattern of cerebrospinal fluid (CSF) biomarkers (Table S1 in Supplementary Material). Although we cannot completely dismiss the notions that the pathogenesis of ADAD and LOAD are distinct and that findings from ADAD do not apply to LOAD, investigation of families with ADAD have contributed enormously to our understanding of AD. Finding mutations that cause ADAD identified key molecules in the disease process (5–9). Transgenic mice expressing human ADAD mutations revolutionized the field and have been used to examine almost every aspect of the disease (10). Recently, studies of CSF and brain imaging biomarkers have helped establish the time course of AD-related brain changes in individuals affected by ADAD, especially during the preclinical stage, prior to the appearance of cognitive symptoms (11, 12). Furthermore, after the failure of numerous drug trials to halt, slow, or reverse cognitive decline in symptomatic individuals with LOAD, clinical trials are now utilizing the unique nature of ADAD and the data derived from these families to design prevention trials for AD dementia in both ADAD mutation carriers (MCs) and individuals at risk for LOAD, while they are still asymptomatic (13, 14). Just as Ms. D’s genetic misfortune benefited the entire field of AD research, it is likely that ADAD patients will lead us to better treatments for all people afflicted by this disease.

## Epidemiology

Alzheimer disease is the most common cause of dementia and, in the United States, affects ~4.7 million individuals aged 65 and older (15). Less than five percent of AD patients develop symptoms before age 65 and are classified as having early onset Alzheimer disease (EOAD) (16). Even rarer are the <1% of AD patients who carry mutations that cause ADAD with 100% penetrance who are distributed world-wide. Carriers of ADAD mutations typically develop symptoms of dementia in their 30s to 60s, depending on their specific gene mutation and the age of onset within their family (17, 18). Much of our current knowledge about ADAD and biomarkers of ADAD comes from two large studies: the multi-center, international Dominantly Inherited Alzheimer Network (DIAN) cohort, and the Alzheimer’s Prevention Initiative (API) cohort that studies a large pedigree living in the state of Antioquia in Colombia, South America. The DIAN cohort includes carriers and non-carrier (NC) family members with many different ADAD mutations, while the Colombian kindred is likely descended from a single individual (19) and carries the E280A mutation in the *PSEN-1* gene.

## Clinical Features

Regardless of whether patients develop symptoms of AD before age 65 (EOAD) or after age 65 (LOAD), the typical first symptom of brain dysfunction is progressive episodic memory loss that slowly worsens over years (20). However, about 30–40% of patients with early symptom onset either from non-familial EOAD or ADAD have an increased frequency of atypical presentations, such as impairments in non-memory domains, including executive, behavioral, language, and visuospatial (21–23). *PSEN-1* MCs have been reported to be more likely to have headaches, myoclonus, gait abnormalities, pseudobulbar affect, and spastic paraparesis (24–26). Some mutations in the gene for amyloid precursor protein (*APP*) cause severe cerebral amyloid angiopathy (CAA), with resultant strokes and brain hemorrhages (27). These clinical features are rarely observed in LOAD.

## Neuropathology

The hallmarks of AD, regardless of the age at dementia onset and its underlying cause (ADAD versus LOAD), are aggregation of the amyloid-β (Aβ) peptide into amyloid plaques and region-specific development of intraneuronal neurofibrillary tangles composed of hyperphosphorylated forms of the microtubule-associated protein, tau (28). AD-affected brains also demonstrate significant neuronal loss and associated neuroinflammation (29–31), although these features are not specific to AD.

In addition to these classic pathologies, some ADAD mutations have been associated with neuropathological abnormalities not typically seen in LOAD. For example, amyloid deposition has been observed in the cerebellum of *PSEN-1* E280A carriers (32), an area not typically affected in LOAD. “Cotton-wool” type plaques that are larger than typical plaques, lack congophilic cores and have few associated dystrophic neurites (33) are often seen in individuals carrying certain *PSEN-1* mutations (34). Some ADAD mutations (notably in *APP*) result in severe CAA, which appears histologically as deposition of Aβ40 in the blood vessel wall. The specific pattern of CAA distribution in the brain depends on the mutation (e.g., Dutch, Flemish, Arctic, Iowa, and Italian) (34).

## Genetics and Pathogenesis

The genetics of ADAD have provided key insights into the molecular pathogenesis of AD. The observation in 1984 that older adults with Trisomy 21, also known as Down syndrome, develop the brain changes of AD suggested that a genetic locus on chromosome 21 might be involved in AD (35). Indeed, the first ADAD mutations were identified in the *APP* gene that resides on chromosome 21, thus implicating amyloid as a key player in AD pathogenesis (5–7, 36). We also now know that duplication of the *APP* locus results in ADAD (37, 38), likely because of amyloid over-production. Following the discovery of *APP* mutations, mutations in *PSEN-1* (8) and the gene for presenilin 2 (*PSEN-2*) (9) were identified and found to increase the amount of the more aggregation-prone Aβ42 compared to Aβ40 (39). Later, it was discovered that presenilin 1 is a critical component of the γ-secretase enzyme complex that cleaves APP to form Aβ (40). To date, 40 mutations in *APP*, 197 mutations in *PSEN-1*, and 25 mutations in *PSEN-2* have been identified that cause ADAD (2).

Since ADAD mutations either increase total Aβ or increase the ratio of Aβ42:Aβ42, amyloid has been hypothesized to be the initiator of AD, an idea described as the “Amyloid Hypothesis” (41). In further support of this hypothesis, a mutation was recently discovered in *APP* that decreases Aβ production and lowers the risk for AD (42). According to this hypothesis, initial deposition of Aβ into amyloid plaques leads to downstream tau-related neuronal pathology (tangles), neuronal injury, and subsequent neuronal death, which is then manifested as cognitive impairment, ultimately culminating in dementia at the end stage of the disease. Data from neuropathological, brain imaging, and CSF biomarker studies in LOAD are consistent with this hypothesis (43–49), but it has only been through study of ADAD that we have a more precise knowledge of the timing of these changes during the early, preclinical (presymptomatic) stage.

## CSF Biomarkers in ADAD

Due to its high prevalence, the majority of AD biomarker studies to date have evaluated individuals with LOAD. CSF levels of Aβ42, tau, and phosphotau181 (ptau) (markers of amyloid, neuronal injury, and tangles, respectively) have stood the test of time in exhibiting both diagnostic and prognostic utility (50). Individuals diagnosed with very mild or mild AD dementia have low levels of CSF Aβ42 (51–54) that inversely correlate with the presence of amyloid as visualized by positron emission tomography (PET) (55–59). Concentrations of CSF tau and ptau are increased in AD and have been shown to positively correlate (albeit to differing degrees) with tangle load at autopsy (52, 53, 60) and regional brain atrophy as defined by magnetic resonance imaging (MRI) (61–64). When paired, the combination of low CSF Aβ42 and high tau/ptau has been shown to be a strong predictor of future cognitive decline in both early symptomatic (very mild dementia or mild cognitive impairment, MCI) and asymptomatic individuals (55, 65–68). However, while such analyses in individuals at risk for LOAD can estimate the risk for decline, they cannot provide the information that is most useful for clinical care – where an individual falls along the pathologic disease cascade or when an individual can expect to develop symptoms of dementia.

In contrast, ADAD provides a unique resource for characterizing changes in CSF biomarkers, especially those that occur long before the onset of dementia. With ADAD families, investigators know if and when an individual will develop dementia. Mutations have 100% penetrance, allowing investigators to know with certainty that an individual will develop AD. Furthermore, within a given family, the age of dementia onset remains fairly consistent, allowing researchers to calculate an estimated number of years until symptom onset (EYO). The EYO construct permits evaluation of biomarker concentrations as a function of where along the disease trajectory an individual falls, independent of the actual age of dementia onset of their parent (17). Using ADAD families, studies can examine biomarker levels in MCs and NCs at distinct time points throughout the course of the disease, including the preclinical AD interval many years prior to dementia onset. However, the low prevalence of ADAD has historically created difficulties in evaluating CSF biomarkers in these families. Most early studies analyzed CSF from fewer than 10 MCs (69–71) (Table 1), and with the exception of those evaluating the large Columbia kindred (*PSEN-1* E280A) (12, 72), most have pooled together carriers of different mutations. Despite the relatively small sample sizes and potential heterogeneity caused by pooling together individuals with different mutations, the pattern of CSF biomarker changes seen in ADAD MCs is remarkably similar to that observed in LOAD, namely, reduced levels of CSF Aβ42 and elevated levels of tau and ptau (Table 1; Table S1 in Supplementary Material). The one exception is very young MCs (in their 20s, about 25 years prior to AD symptom onset), who have elevated CSF Aβ42 (72). This was hypothesized to reflect over-production of CSF Aβ42 in ADAD MCs, which has more recently been confirmed directly in kinetic studies (73).

**Table 1:** **Studies examining CSF biomarkers in participants with autosomal dominant Alzheimer disease**.

Study	Mutation(s)	Aβ42	Tau	pTau	Comments
Moonis et al. (69) 6 MC 6 Unrelated controls	*PSEN-1* C410Y, *PSEN-1* P242H, and R352H	↓	N.S.	Not tested	MC EYO −8 ± 3
Ringman et al. (74) 20 MC 9 NC	*PSEN-1* A431E, *PSEN-1* L235V, *PSEN-1* G206A, *APP* V717I	↓ (trend)	↑	↑	MC EYO ~−12 NC EYO −9 ± 12
Fortea et al. (70) 8 MC 5 NC	*PSEN-1* L286P, *PSEN-1* M139T	↓ (trend)	N.S.	N.S.	MC EYO −6 ± 10 NC EYO −7 ± 9
Scholl et al. (71) 4 MC 7 Unrelated controls	*APP* KM670/671 NL, *APP* E693G, *PSEN-1* H163Y	↓	↑	↑	
Reiman et al. (72) 10 MC 10 NC	*PSEN-1* E280A (API)	↑	N.S.	N.S.	MC EYO ~−25 NC EYO ~−26
Ringman et al. (75) 13 MC 5 NC	*PSEN-1* A431E, *PSEN-1* L235V, *PSEN-1* S212Y, *APP* V717I	↓	↑	↑	MC EYO −12 ± 10 NC EYO −6 ± 14
Bateman et al. (11) 88 MC 40 NC	Many (DIAN)	↓ at EYO −10 and closer to EAO	↑ at EYO −15 and closer to EAO	Not shown	
Thordardottir et al. (76) 10 MC 12 NC	*APP* KM670/671 NL, *APP* E693G, *PSEN-1* H163Y, *PSEN-1* I143T	↓	↑	↑	MC EYO −7 ± 9 NC EYO −7 ± 12
Fleisher et al. (12) 32 MC 22 NC	*PSEN-1* E280A (API)	↓ at EYO −25 and closer to EAO	↑ at EYO −20 and closer to EAO	↑ at EYO −18 and closer to EAO	

*API, Alzheimer’s Prevention Initiative; *APP*, amyloid precursor protein; DIAN, Dominantly Inherited Alzheimer Network; EAO, estimated age of symptom onset; EYO, estimated years to symptom onset; MC, mutation carrier; NC, mutation non-carrier (typically first-degree relative of MC); N.S., not significant; *PSEN-1*; presenilin 1*.

*Numbers in parentheses refer to associated reference*.

**Figure 1:**
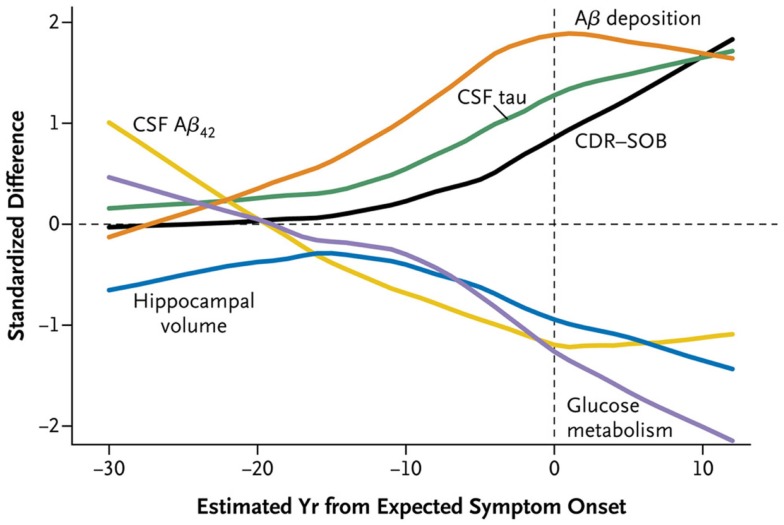
**A time course of changes in ADAD mutation carriers versus non-carriers**. Cross-sectional data obtained in the DIAN cohort demonstrates that CSF Aβ42 (yellow) declines as Aβ deposition increases as shown by amyloid PET imaging (orange). CSF tau (green) increases as hippocampal volume (blue) and glucose metabolism as shown by FDG PET (purple) decreases. CDR-SOB (Clinical Dementia Rating-Sum of Boxes) (black), which quantifies clinical symptoms of dementia, increases (indicating worse performance) relatively late in the disease course. Reproduced with permission from Bateman et al. (11).

The larger DIAN and API studies have permitted analysis of CSF and imaging biomarkers in greater numbers of both asymptomatic and symptomatic individuals that span a wide range of EYOs, thus allowing conclusions to be drawn regarding the timing of such biomarker changes during the preclinical period (Figure 1). Results from cross-sectional analyses demonstrate higher levels of CSF Aβ42 in MCs compared to NCs very early in the disease process (~20–30 years prior to estimated symptom onset, EYO −20 to −30), which then drop with disease progression, becoming significantly lower than NCs ~10–20 years prior to symptom onset (~EYO −10 to −20) (11, 12, 72, 77). These low levels then begin to plateau with the development of cognitive symptoms. After Aβ42 levels begin to drop, levels of tau and ptau in MCs become significantly higher than NCs (~EYO −15), and then continue to increase with disease progression. However, a recent study of within-person change in biomarkers in a small sub-cohort of DIAN participants with longitudinal biomarker data has shown that although levels of CSF tau and ptau increase in MCs during the preclinical (asymptomatic) phase, levels stabilize or decline over time in individuals who are symptomatic (77). Similar patterns were observed in levels of visinin-like protein 1 (VILIP-1) (77), a neuronal calcium sensor protein that is a marker of neuronal injury/death (78). Consistent with this pattern, a previous report of a single asymptomatic ADAD (*APP* V717I) MC showed substantial increases in tau and ptau over a 4- to 5-year period very early in the disease process (~EYO −19 to −14) (79), whereas a longitudinal decrease (or a lack of increase) in ptau was reported in a small Japanese cohort (*n* = 4) of symptomatic *PSEN1* MCs (80). Although not often discussed, results consistent with these changes in the trajectories of neuronal injury-related markers have been reported in LOAD (81–83).

Although this general model is consistent with data obtained from cross-sectional studies in LOAD (49, 84–86) and suggests a common pathophysiology for AD due to mutations and the much more common “sporadic” form, the longitudinal data from DIAN supports a model that incorporates an eventual slowing down of the rate of neuronal injury and death as may be indicated by reductions in these markers. It is also possible that the later decreases during the symptomatic phase may reflect fewer neurons left to contribute to the pool of CSF tau/ptau/VILIP-1. If corroborated in additional cohorts, this reversing pattern of marker change will likely have an impact on the definition of a positive neurodegenerative biomarker outcome in clinical trials, especially during the symptomatic phase. For example, an effective therapy may only slow the rate of increase in injury markers in individuals who are in the preclinical phase, but stabilize or decrease the rate of change in injury markers later in the disease. Confirmation of such patterns awaits evaluation of biomarker trajectories in clinical trials.

## Use of ADAD in Clinical Trial Design

Many clinical trials in symptomatic individuals with LOAD have failed to meet their clinical endpoints of delaying, halting, or reversing cognitive decline. One possibility proposed to explain this failure is that therapies must be delivered earlier, in individuals known to have underlying AD pathology, but before significant symptoms are manifest (87). However, there are several challenges associated with the design and implementation of such “prevention trials,” including identifying asymptomatic participants with known underlying AD pathology and who are at a point in their disease trajectory when they are close to becoming symptomatic. Although CSF and imaging biomarkers are currently being used in clinical trials to confirm underlying amyloid pathology in individuals at risk for developing LOAD (http://www.nia.nih.gov/alzheimers/clinical-trials/), the onset of dementia in LOAD is characteristically difficult to predict, even in individuals who are biomarker-positive. As a result, large numbers of participants are required in order to provide adequate statistical power to show a potential drug effect. In contrast, since ADAD is fully penetrant and the time until onset of dementia symptoms in MCs can be predicted with relatively high precision, fewer trial participants are required to demonstrate treatment efficacy within a suitable timeframe. Two such prevention trials in ADAD are currently underway; the DIAN-Trials Unit (DIAN-TU) and API, both of which are testing monoclonal antibodies directed against various forms of Aβ (13, 14).

Another possibility to explain the failure of previous clinical trials in LOAD is that the drug did not engage its purported target. Given the compelling data from observational biomarker studies of ADAD (Table 1), biomarkers can serve as meaningful endpoints to verify target engagement even before the possible appearance of significant cognitive effects. To this end, the DIAN-TU has defined biomarkers as the primary endpoint [amyloid PET or CSF Aβ, with CSF tau(s) as downstream targets], with the trial design transitioning to a cognitive endpoint only for those drugs shown to have properly engaged their pathologic targets (14, 88, 89). CSF biomarkers are also being used as exploratory measures in the API trial (13) and the Anti-Amyloid Treatment in Asymptomatic Alzheimer’s (A4) prevention trial in LOAD (90).

## Conclusion

Although there are some differences in the pathology and clinical expression in ADAD compared to LOAD (Table S1 in Supplementary Material), studies of ADAD have provided critical insight that has propelled our knowledge and investigation of all forms of AD. Investigators have proposed the relative timing of biomarker changes in LOAD (48, 49), but these hypotheses cannot yet be empirically verified because we do not know *a priori* when individuals with LOAD will develop symptoms. Because the EYO is known in ADAD cases, data-based models of AD can be generated (Figure 1) (11, 12). Curves representing changes in CSF and imaging biomarkers over the disease course in ADAD can be superimposed on curves of cognitive function, resulting in a detailed road map of AD pathologic processes. These analyses confirm that AD brain changes begin to develop over two decades before the onset of dementia. Now, as researchers work to develop drugs that prevent dementia associated with AD pathology, they are using ADAD to accelerate clinical trials (13, 14). It would be appropriate if ADAD, which represents <1% of all AD but has provided so much insight into the disease, leads to a drug that ultimately prevents all forms of AD.

## Author Contributions

SS and AF were involved in all aspects of preparing, writing, and editing the manuscript.

## Conflict of Interest Statement

The authors declare that the research was conducted in the absence of any commercial or financial relationships that could be construed as a potential conflict of interest.

## Supplementary Material

The Supplementary Material for this article can be found online at http://journal.frontiersin.org/article/10.3389/fneur.2015.00142
