# Supplementary material for: Autosomal Dominant Alzheimer Disease: A Unique Resource to Study CSF Biomarker Changes in Preclinical AD
Source: Front Neurol. 2015 Jun 29;6:142. doi: 10.3389/fneur.2015.00142 (PMC4483518; doi:10.3389/fneur.2015.00142)
Supplement: Supplementary file 1 [file Table_1.PDF]

Supplementary Material

Autosomal dominant Alzheimer disease: A unique resource to study CSF biomarker changes in preclinical AD

Suzanne E. Schindler[1] and Anne M. Fagan[1]

[1]Department of Neurology, The Knight Alzheimer's Disease Research Center and the Hope Center for Neurological Disorders, Washington University School of Medicine, St. Louis, MO, USA

*** Correspondence:** Anne M. Fagan, Department of Neurology, Washington University School of Medicine, Campus Box 8111, 660 South Euclid Avenue, Saint Louis, MO, 63110, USA
fagana@neuro.wustl.edu

Supplemental Table S1. Comparison of autosomal-dominant AD (ADAD) with late-onset AD (LOAD).

Characteristic	ADAD	LOAD
**Prevalence**	<1% of AD cases	>99% of AD cases
**Cause**	Genetic mutations<br>(*APP, PSEN-1, PSEN-2*)	Unknown<br>Major known risk factors<br>(age, *APOE* genotype)
**Clinical features**	Primary: episodic memory impairment<br>Other: executive, behavioral, language, visuospatial<br>Mutation-specific: headache, myoclonus, spastic paraparesis, gait abnormalities, pseudobulbar affect, brain hemorrhages (due to CAA)	Primary: episodic memory impairment<br>Other: executive, behavioral, language, visuospatial
**Age of symptom onset**	<65 years (typically 30-60 years)<br>Predictable within families	≥65 years<br>Sporadic, not predictable
**Neuropathology**	Plaques and tangles in all,<br>CAA in some (especially *APP*),<br>"cotton wool" plaques in some (especially *PSEN-1*)	Plaques and tangles in all,<br>CAA in some
**CSF Aβ42**	↓~50%<br>↑ very early (~30 years prior to symptoms)	↓~50%
**CSF tau and ptau181**	↑ ~100%	↑ ~100%
**Plasma Aβ42 or Aβ42/Aβ40 ratio**	Increased	Not increased, variable
**Amyloid PET imaging**	Prefrontal, precuneus, posterior cingulate, striatum, sometimes cerebellum	Prefrontal, precuneus, posterior cingulate, sometimes striatum
**Volumetric MRI**	↓ hippocampus, temporo-parietal cortex	↓ hippocampus, temporo-parietal cortex
**FDG PET**	Temporo-parietal hypometabolism	Temporo-parietal hypometabolism

Aβ, amyloid-β; ADAD, autosomal-dominant Alzheimer disease; *APOE*, apolipoprotein E; *APP*, amyloid precursor protein; CAA, cerebral amyloid angiopathy; CSF, cerebrospinal fluid; FDG, fluorodeoxyglucose; LOAD, late-onset Alzheimer disease; MRI, magnetic resonance imaging; PET, positron emission tomography; *PSEN-1*, presenilin-1; *PSEN-2*, presenilin-2
